# Supplementary figures and images for: The Epigenome of Evolving Drosophila Neo-Sex Chromosomes: Dosage Compensation and Heterochromatin Formation
Source: PLoS Biol. 2013 Nov 12;11(11):e1001711. doi: 10.1371/journal.pbio.1001711 (PMC3825665; doi:10.1371/journal.pbio.1001711)

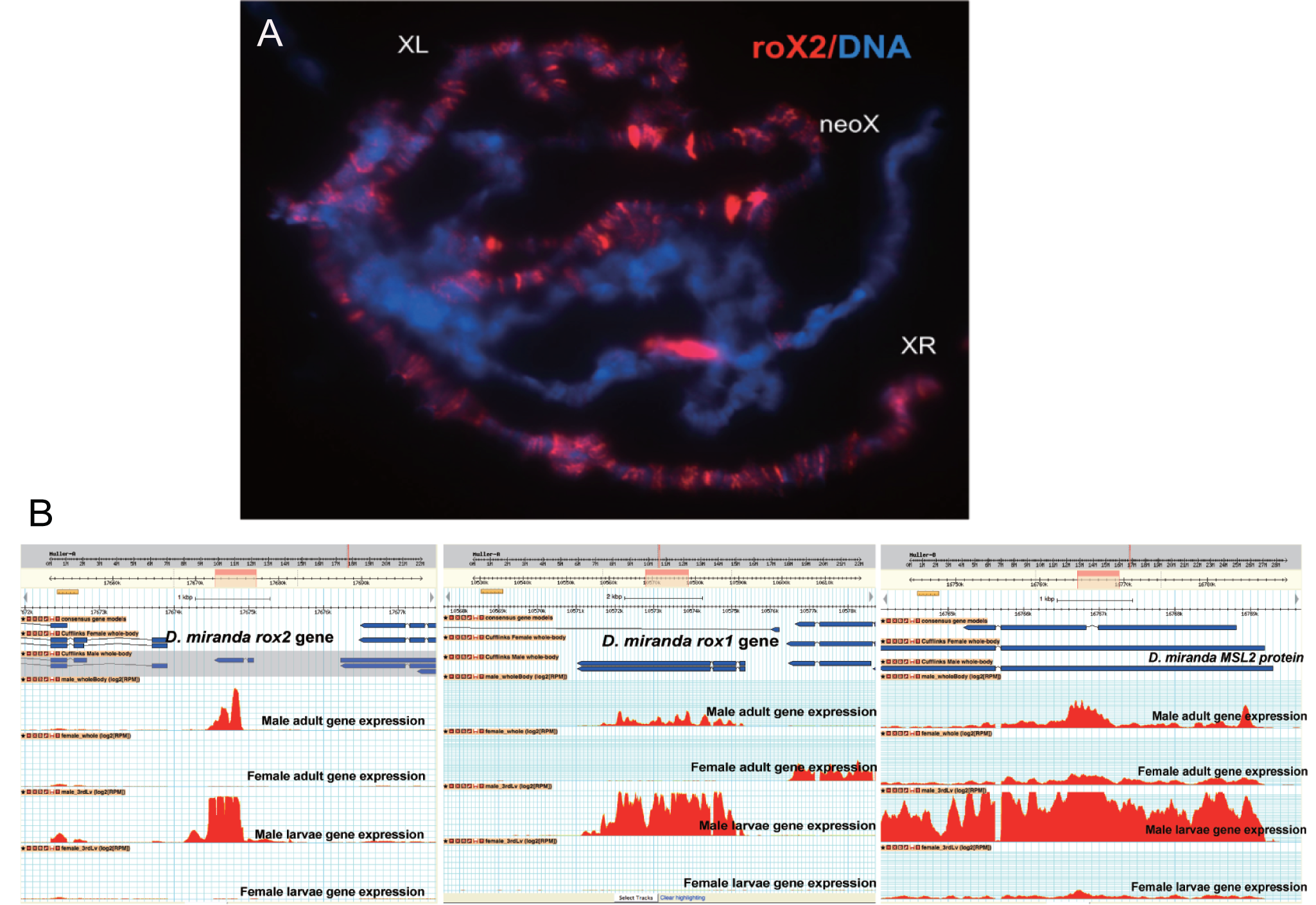

Supplement: Figure S1 — Male-specific targeting and expression of the MSL-complex in D. miranda . (A) roX2 RNA-FISH of D. miranda male salivary glands. We cloned the roX2 gene and performed RNA-FISH, using a similar protocol as described in [60]. (B) Male-specific expression of MSL2, roX1, and roX2. (TIF) [file pbio.1001711.s002.tif]

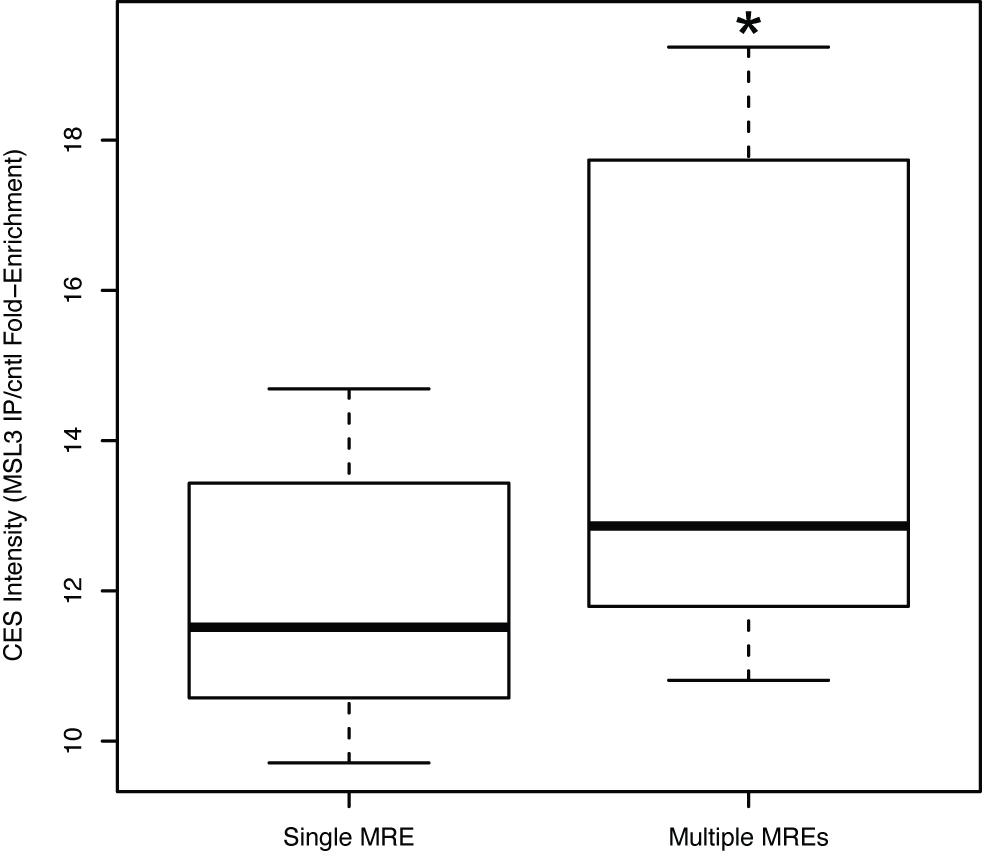

Supplement: Figure S2 — Intensity of MSL-binding versus number of MRE motifs found at CES. CES with multiple MREs show significantly more MSL-binding, than CES with single MREs (one-tailed Wilcoxon test p = 0.038). (TIF) [file pbio.1001711.s003.tif]

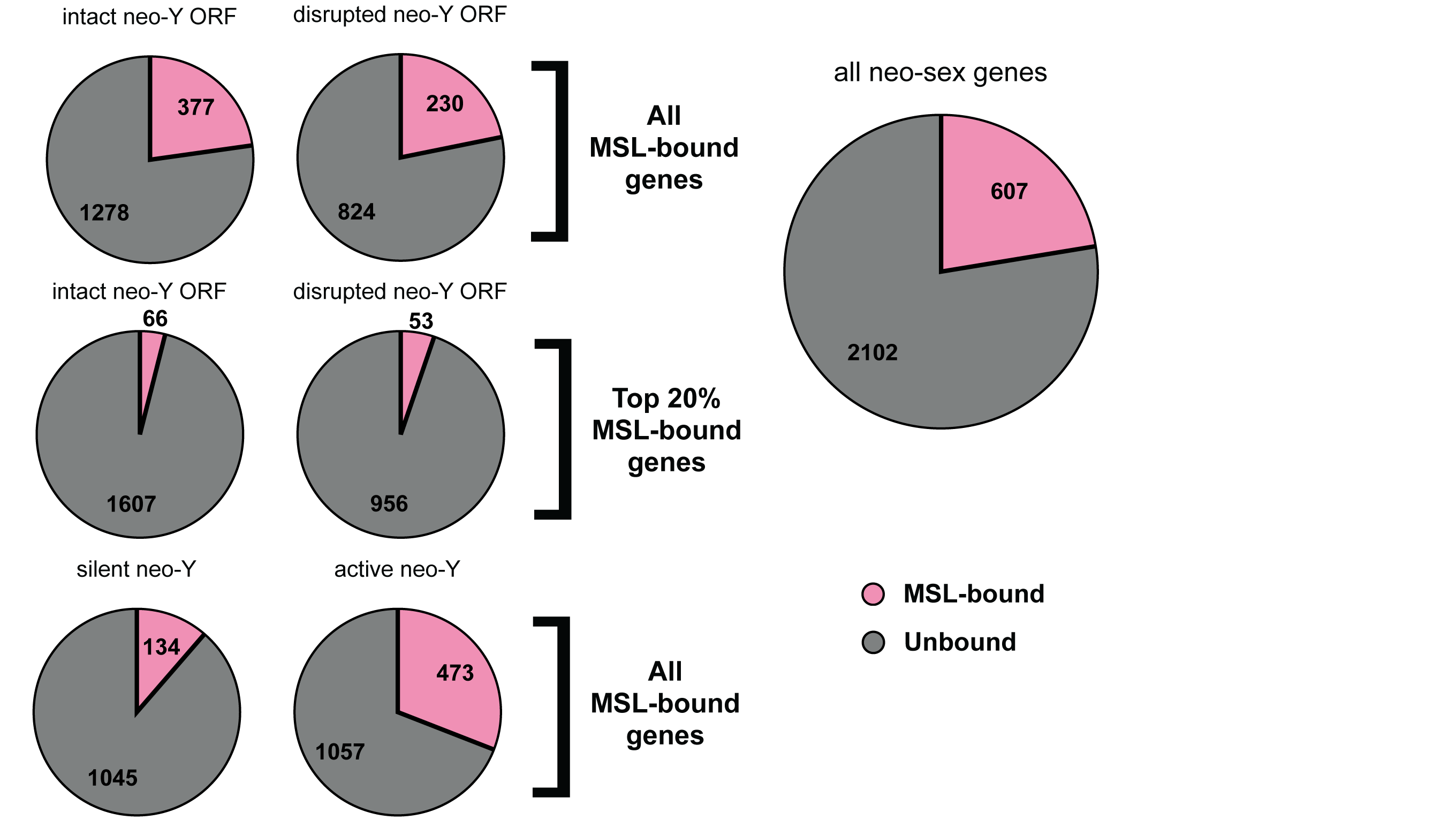

Supplement: Figure S3 — Dosage compensation and neo-Y degeneration. Genes that are targeted by the MSL complex on the neo-X are shown in pink, and genes that are not bound by MSL are shown in grey. (A) The proportion of MSL-bound genes does not differ between neo-X genes whose neo-Y homologs are potentially functional (intact neo-Y ORF) versus those whose neo-Y homologs are non-functional (disrupted neo-Y ORF). Genes that are transcriptionally silent on the neo-Y are less likely to be dosage compensated on the neo-X, while actively transcribed neo-Y genes are more often dosage compensated. (TIF) [file pbio.1001711.s004.tif]

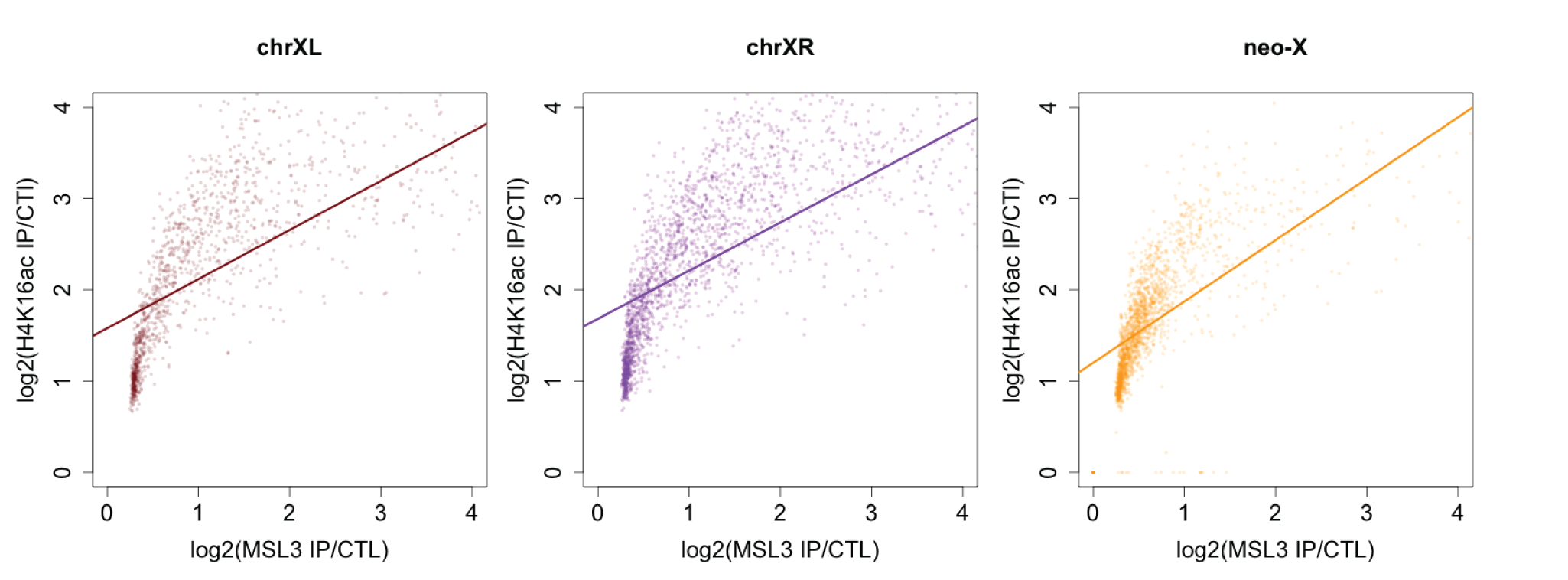

Supplement: Figure S4 — MSL3 enrichment level is significantly correlated with that of H4K16ac. Shown are dot plots of log2 read depth ratios of ChIP-seq versus input control along the gene body for MSL3 and H4K16ac chromatin marker on different X chromosomes, which significantly correlate with each other (R-square = 0.47–0.49, p-value<2.2e–16). (TIF) [file pbio.1001711.s005.tif]

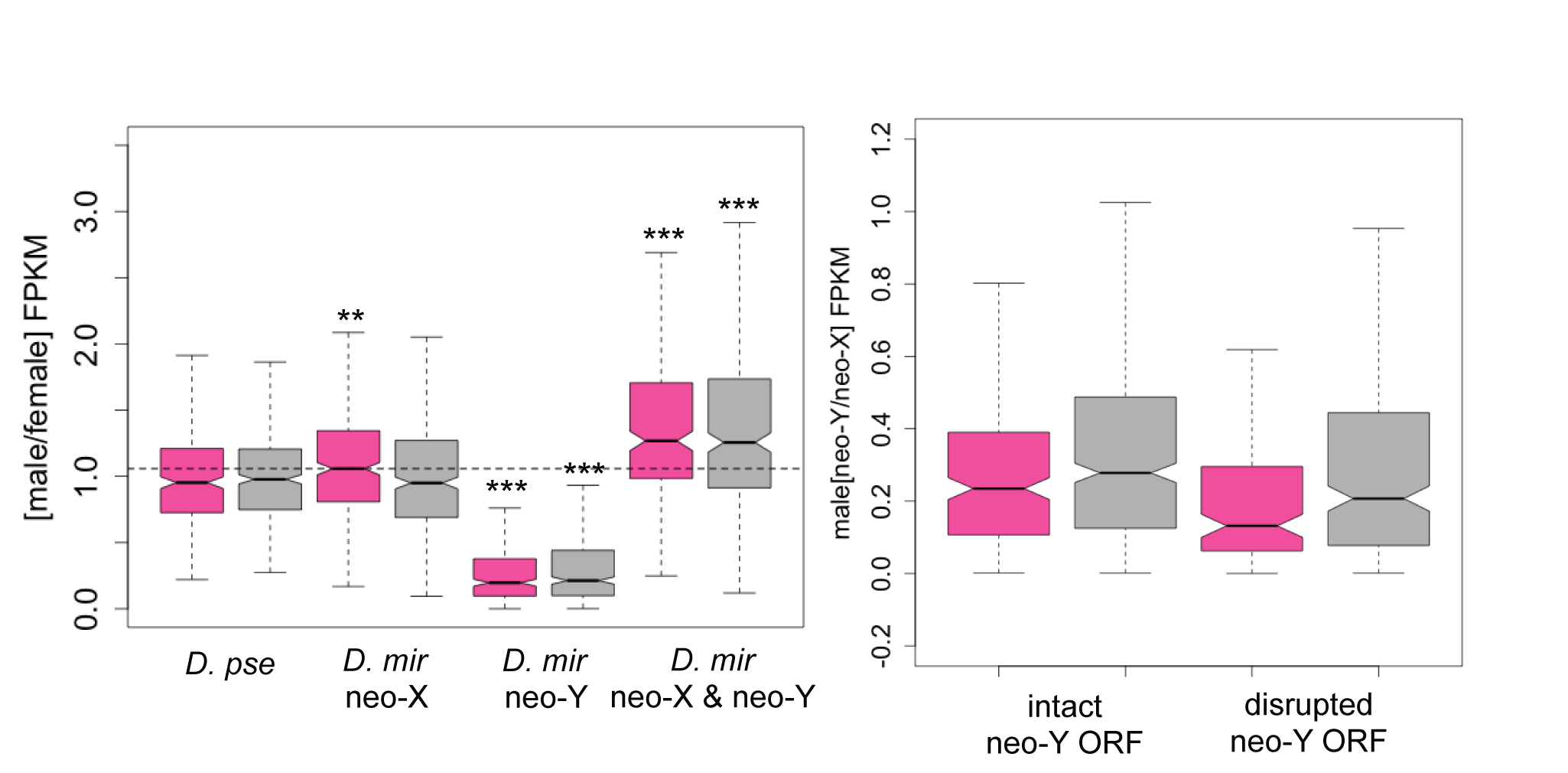

Supplement: Figure S5 — Pattern of dosage compensated genes that are defined by MSL binding only. We observe similar patterns as in Figure 4C and Figure 4D if we define dosage compensated genes on the neo-X only by significant MSL binding. (TIF) [file pbio.1001711.s006.tif]

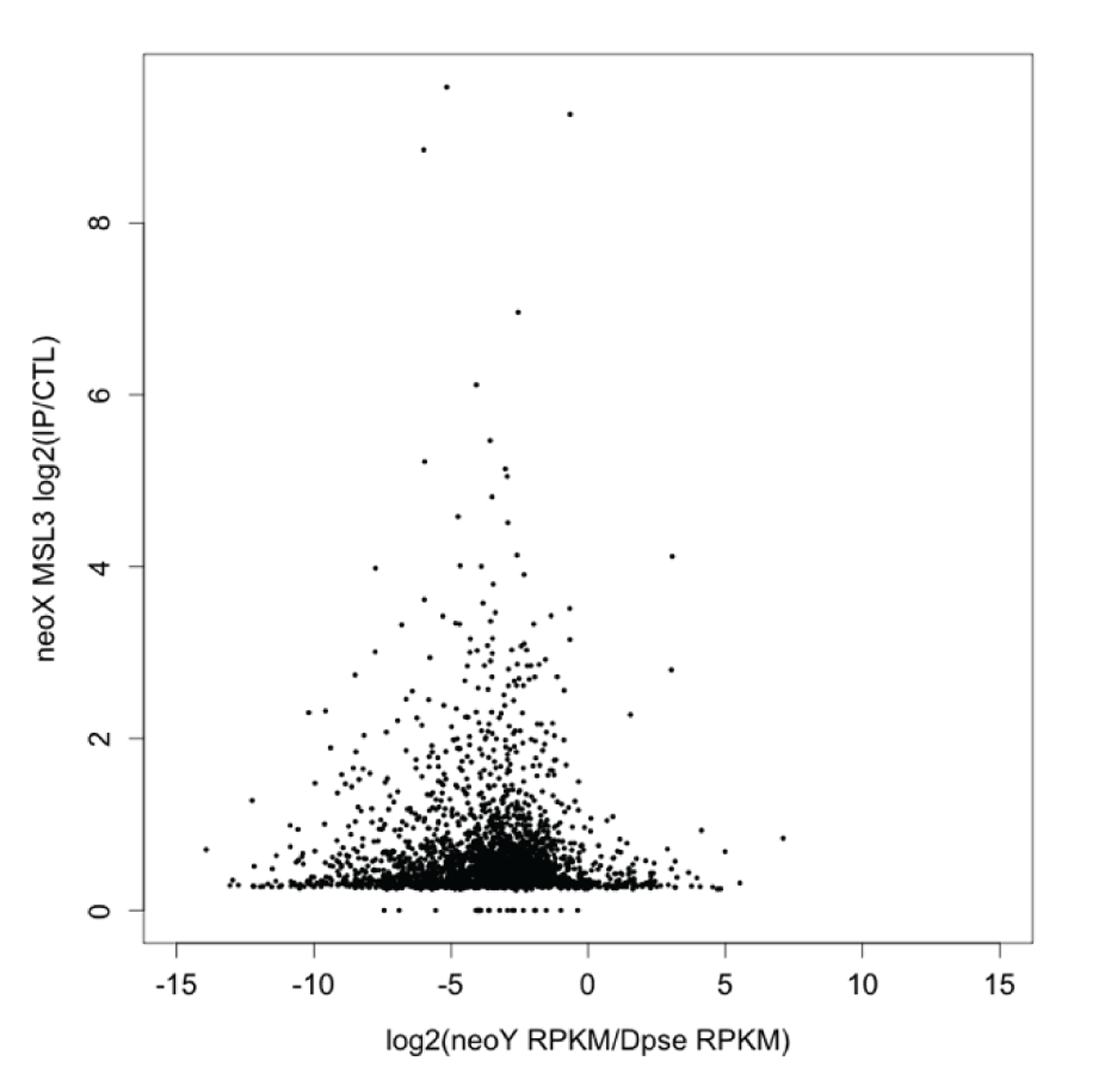

Supplement: Figure S6 — No correlation of neo-Y downregulation versus neo-X dosage compensation. The x-axis shows reduction of neo-Y expression level measured as the log2 ratio of neo-Y gene specific FPKM values versus those of D. pseudoobscura orthologs against the MSL-binding enrichment ratio of their corresponding neo-X genes. (TIF) [file pbio.1001711.s007.tif]

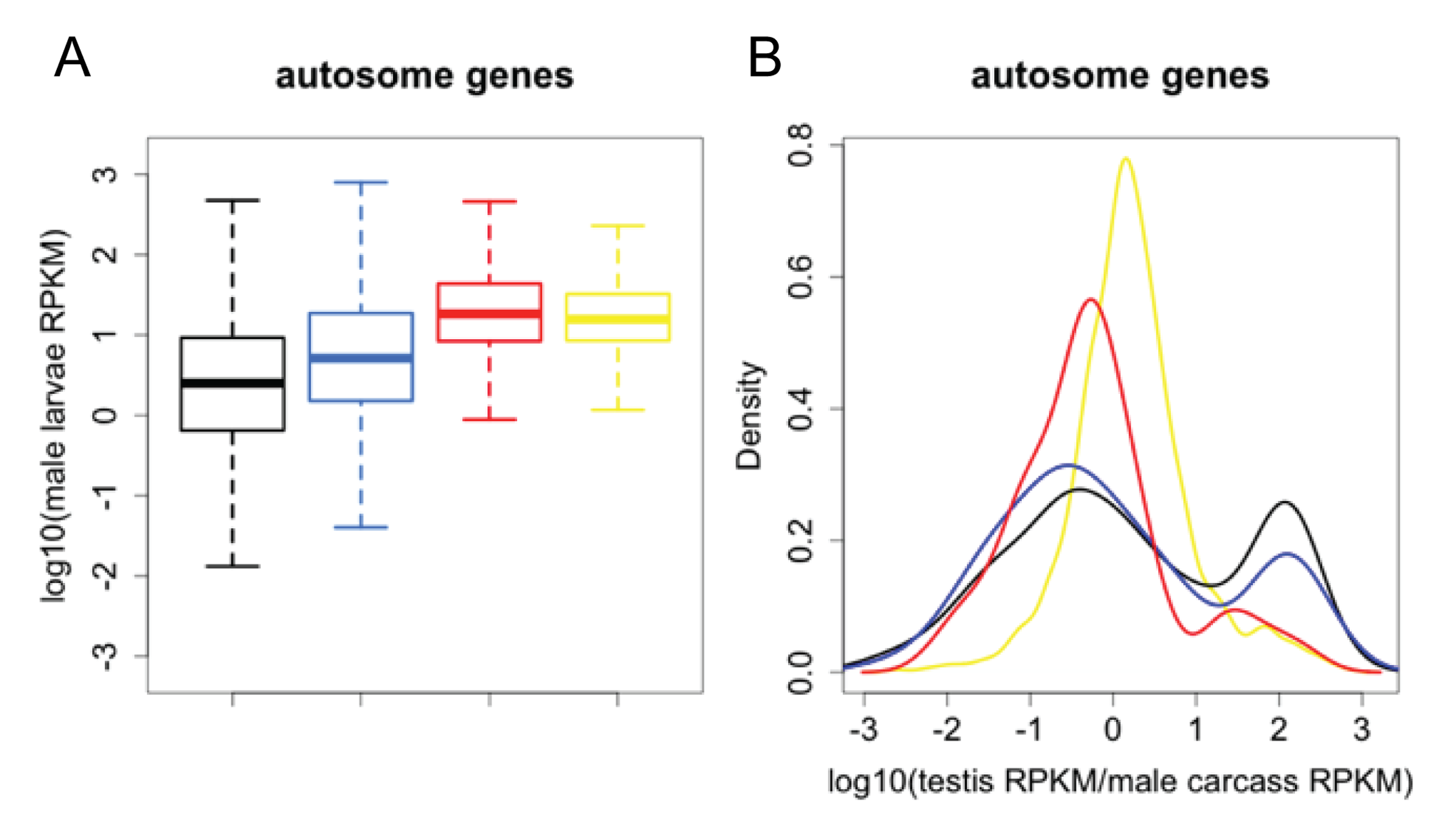

Supplement: Figure S7 — Gene expression patterns for autosomal genes in D. miranda , classified by their different chromatin types defined in D. melanogaster . (A) We find characteristic D. miranda gene expression patterns of each chromatin type that is similar to that of D. melanogaster (i.e., reduced gene expression in repressive “black” or “blue” chromatin, and higher gene expression in active “red” or “yellow” chromatin). (B) Genes in black and blue chromatin are more tissue-specific (measured by testis-specificity in D. miranda), consistent with their patterns of tissue-specific expression in D. melanogaster. These consistent expression patterns between species suggest that we can approximate the D. miranda ancestral chromatin types by their D. melanogaster orthologs. (TIF) [file pbio.1001711.s008.tif]

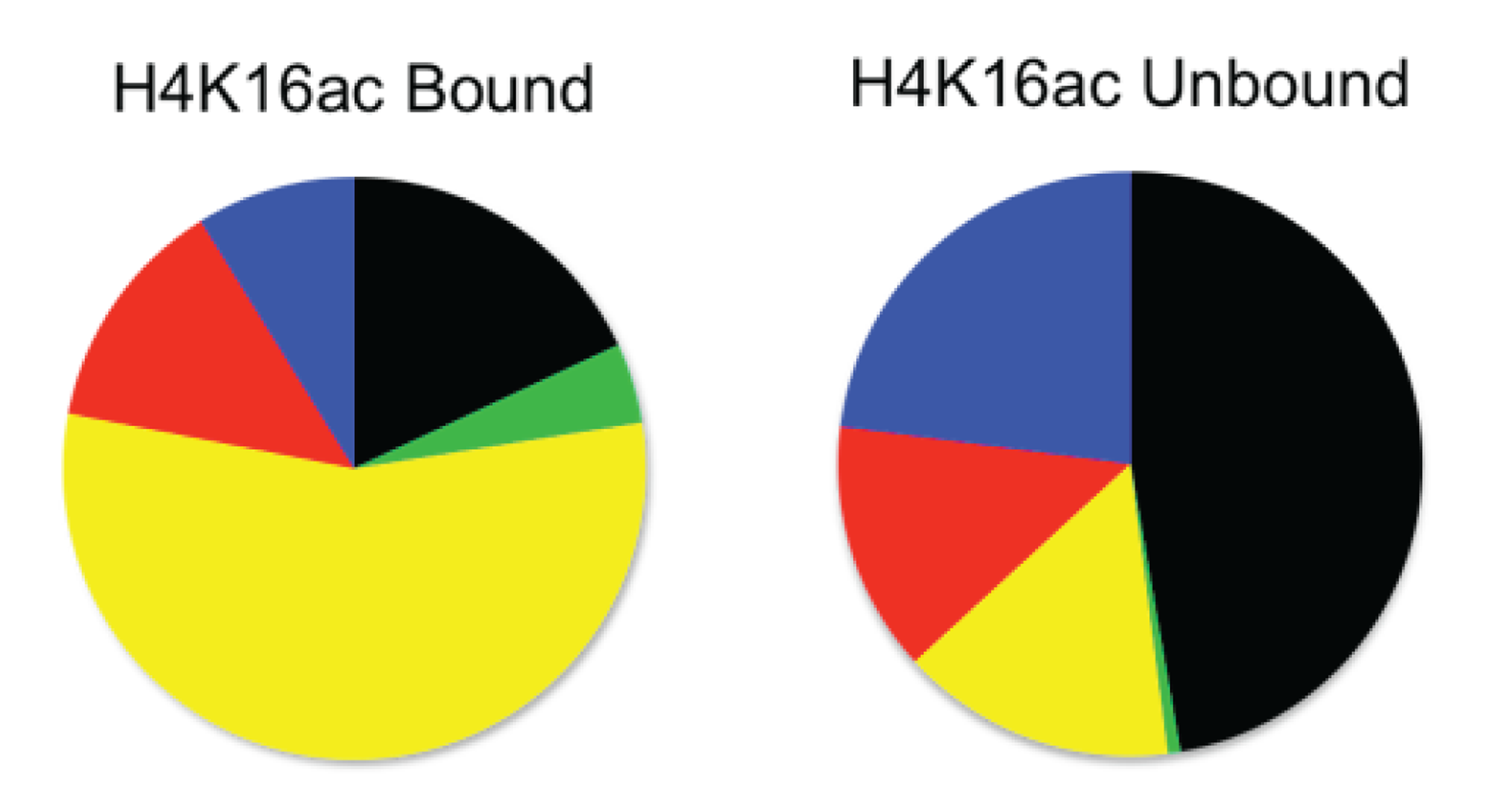

Supplement: Figure S8 — Ancestral chromatin states of H4K16ac bound/unbound genes on chrXR. ChrXR (the Muller D element) is another young X chromosome that originated around 15 MY ago in an ancestor of D. miranda and D. pseudoobscura, and has evolved full dosage compensation. Dosage compensated genes on XR (defined as those bound by H4K16ac chromatin marks) are enriched for genes within an active chromatin state (“yellow” chromatin) in D. melanogaster. (TIF) [file pbio.1001711.s009.tif]

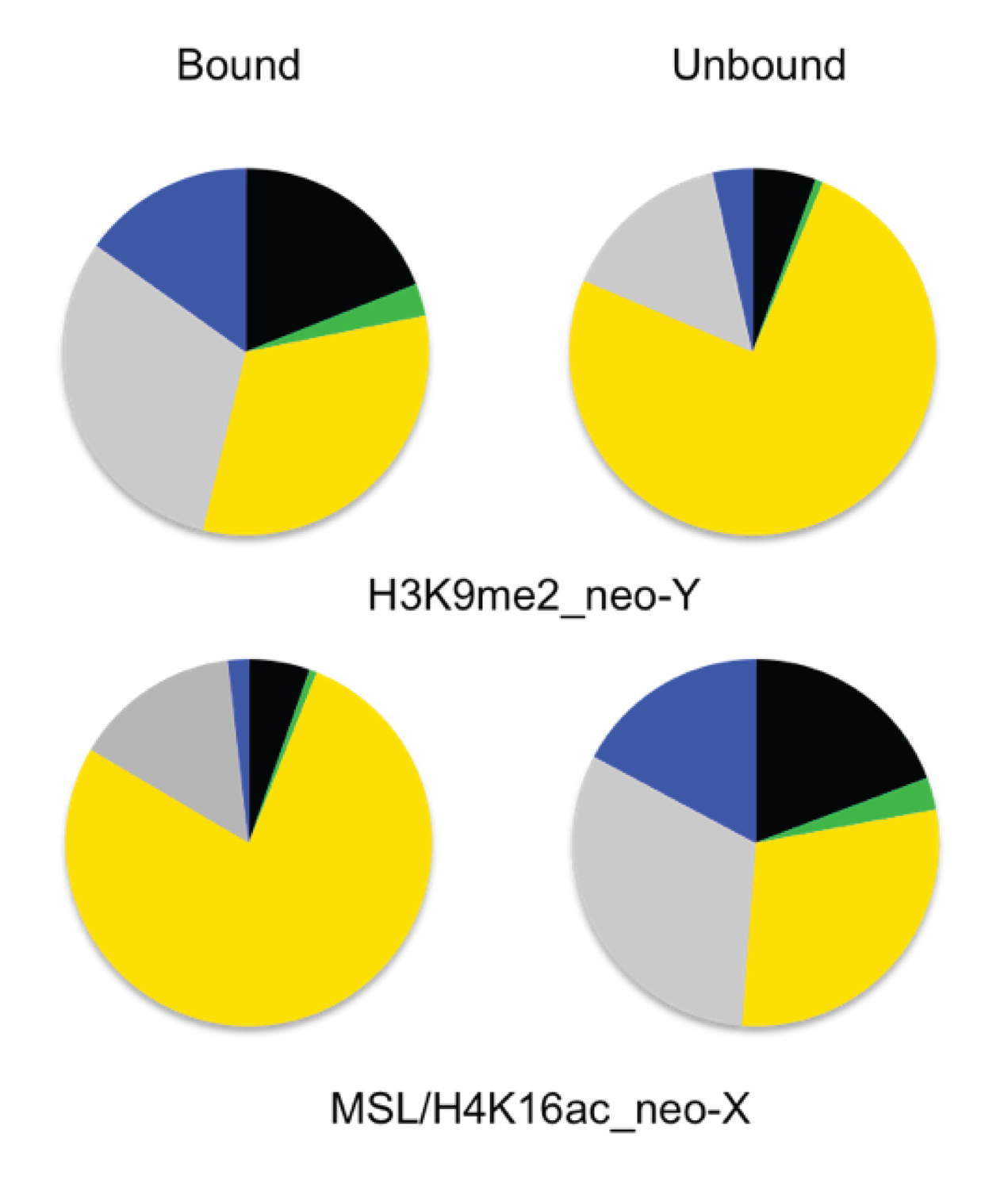

Supplement: Figure S9 — H4K16ac-bound/unbound neo-X genes and H3K9me2-bound/unbound neo-Y genes versus chromatin states of female D. miranda . We approximate the ancestral chromatin states by ChIP-seq data of female D. miranda larvae: blue genes were defined by their characteristic H3K27me3 bound state, green genes by H3K9me2, yellow genes by H3K36me3 and high expression level (FPKM>2), while black genes are not bound by any studied histone markers and show a low expression level (FPKM<2). We grouped the rest of the genes into an unclassified category as grey genes. (TIF) [file pbio.1001711.s010.tif]

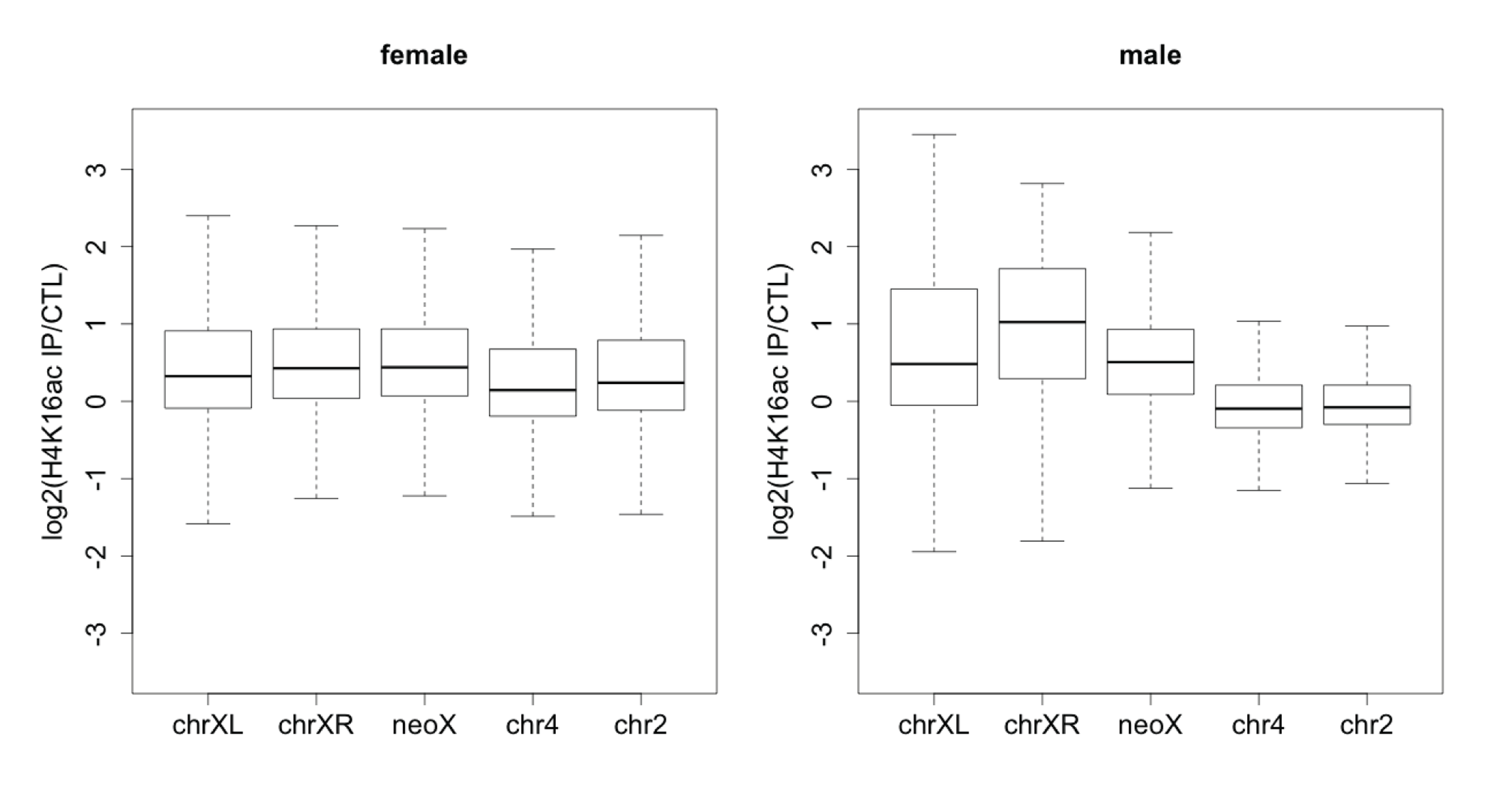

Supplement: Figure S10 — Chromatin structure of sex chromosomes versus autosomes in males versus females. Chromatin structure (as measured by H4K16ac enrichment) is similar between the X and autosomes in females and differs dramatically on the X and autosomes in males of D. miranda. Each boxplot shows log2 read depth ratio of ChIP-seq versus input control along the gene body including the flanking 3 kb regions on a specific chromosome. (TIF) [file pbio.1001711.s011.tif]

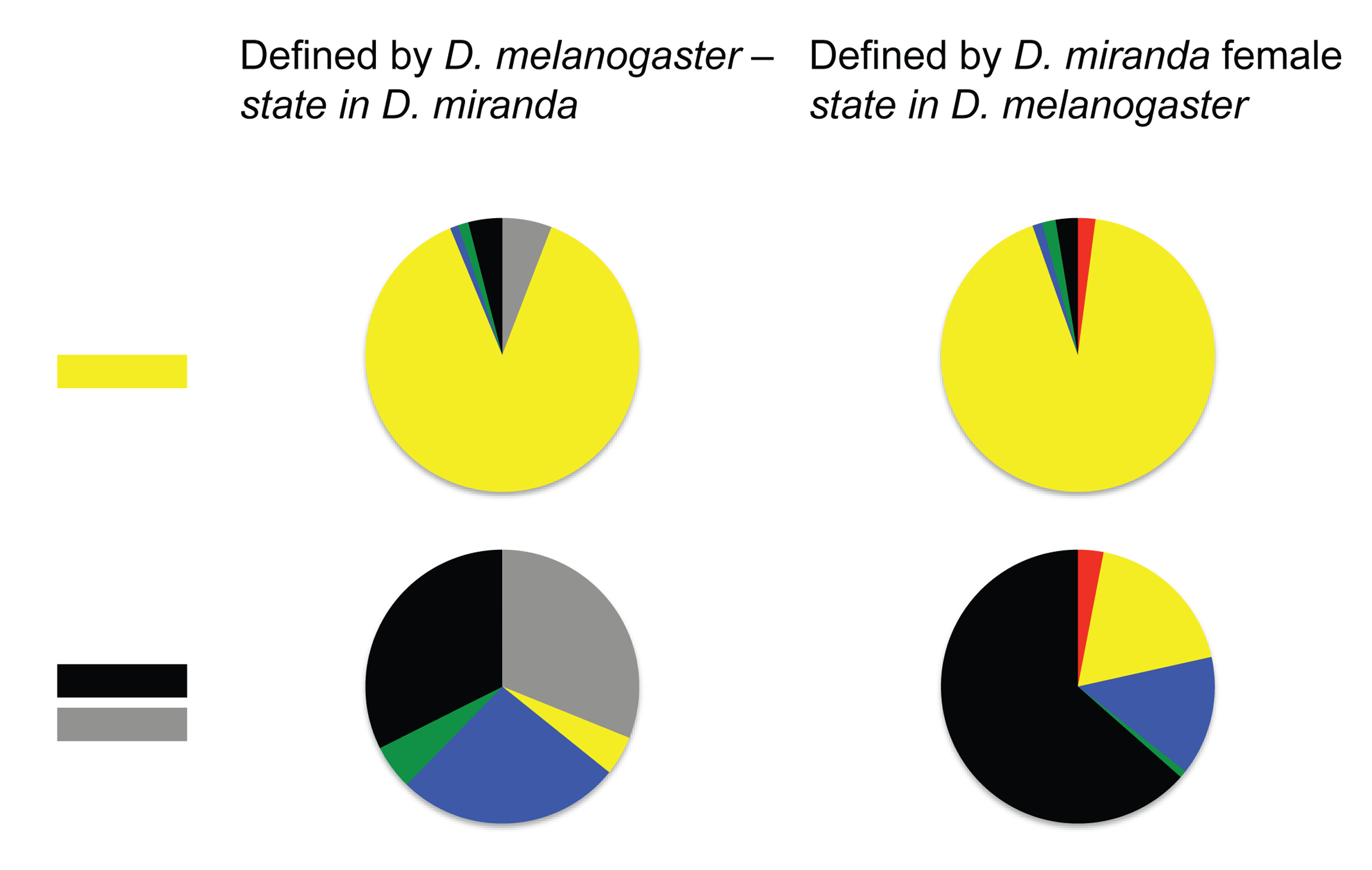

Supplement: Figure S11 — Chromatin states are overall conserved between D. melanogaster and D. miranda females. We define chromatin types either in D. melanogaster, using the classification of [43], or in D. miranda, using the classification described in Figure S9. The pie charts show the composition of a particular type of chromatin defined in one species (active “yellow” chromatin on top; inactive “black” [and “grey” for D. miranda] on the bottom) in the other species. For example, the upper left pie shows the “yellow” genes defined by D. melanogaster and their chromatin type compositions defined using D. miranda female data. Overall, both definitions of active versus repressive chromatin show a high overlap between species, suggesting chromatin types of orthologous genes are relatively conserved. (TIF) [file pbio.1001711.s012.tif]

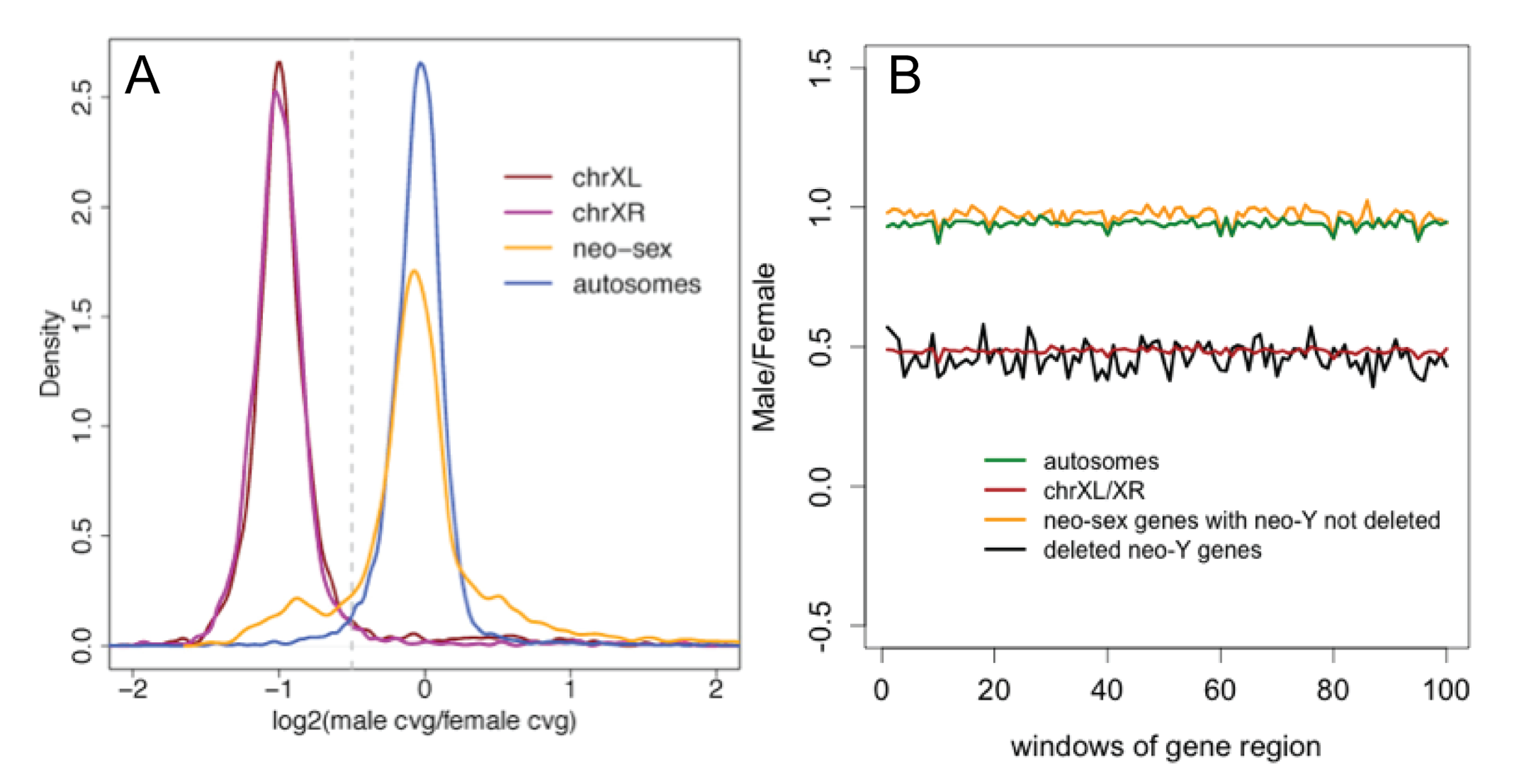

Supplement: Figure S12 — Identification of deleted genes on the neo-Y chromosome. (A) Shown is the histogram of male versus female coverage ratios at exonic regions for all D. miranda genes. A cutoff (dotted line, log2(male/female) = −0.5) separating the distribution of autosomes and X chromosomes was picked to identify genes that are deleted from the neo-Y chromosome. (B) Metagene plot of male/female coverage for different classes of genes (X-linked, autosomal, neo-sex genes with/without deleted neo-Y), across the gene body. (TIF) [file pbio.1001711.s013.tif]

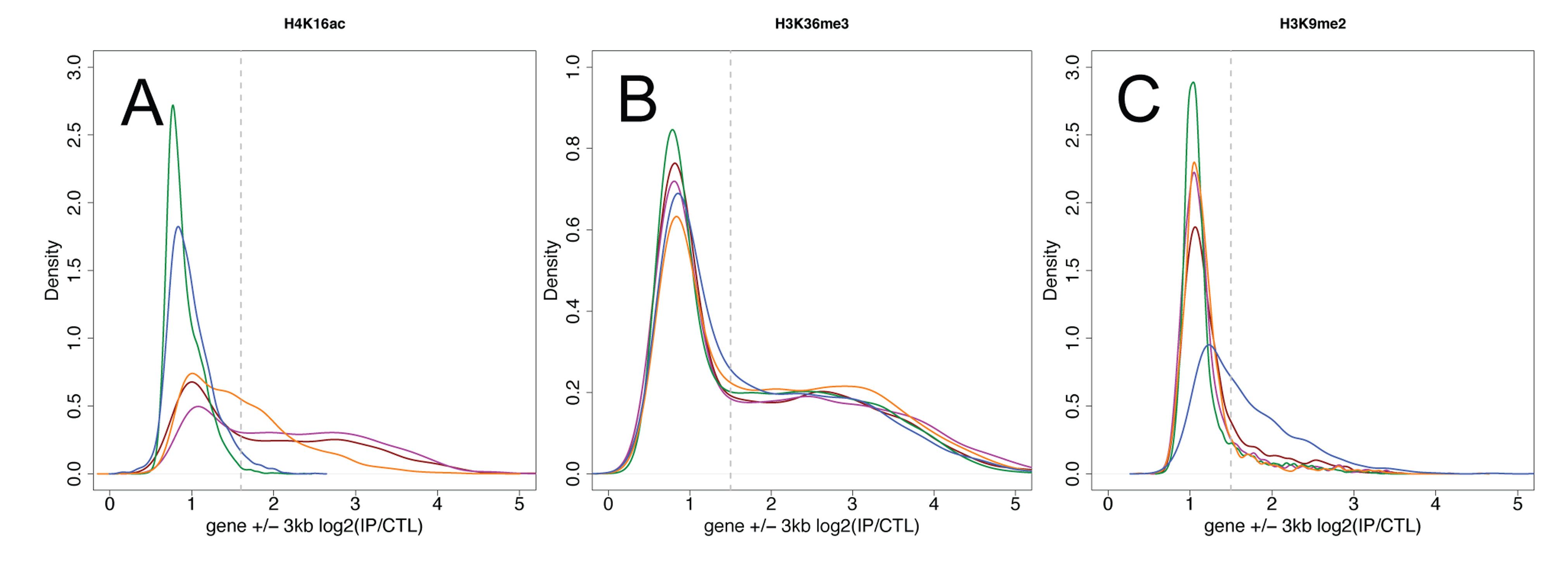

Supplement: Figure S13 — Definition of bound/unbound genes for different chromatin marks. Shown is the histogram of the log2 coverage ratio of ChIP-seq versus input control along the gene body including up/downstream 3 kb regions separately for each chromosome. Autosomes are in green, chrXL in red, chrXR in purple, neo-X in orange, and neo-Y in blue. Cutoffs discriminating bound/unbound genes were chosen where the bimodal distribution is separated for two peaks or sex/neo-sex chromosomes are separated from the autosomes. (TIF) [file pbio.1001711.s014.tif]

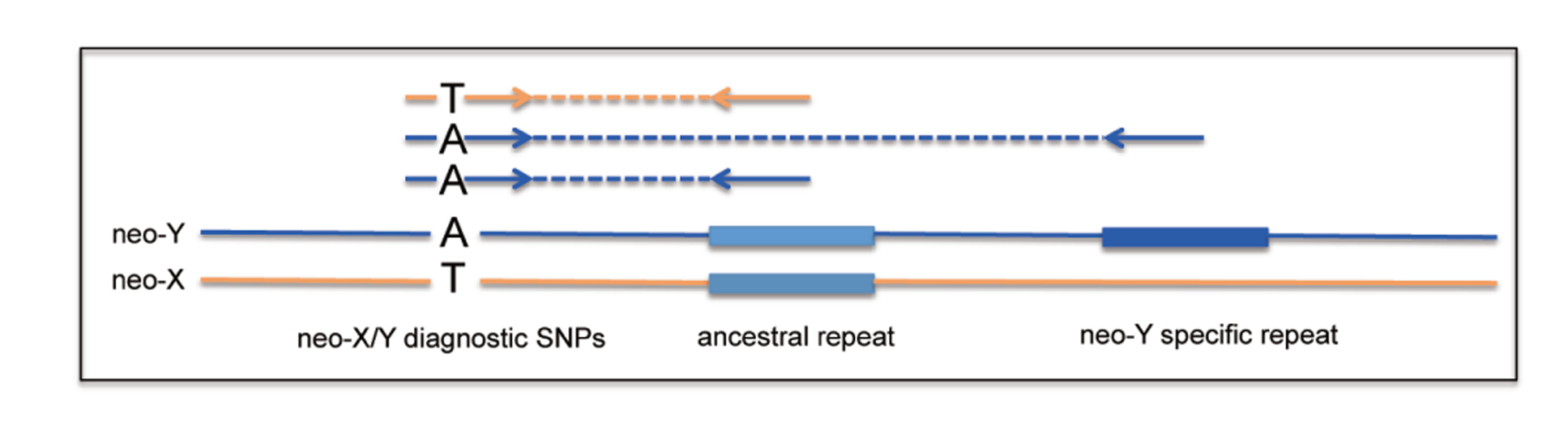

Supplement: Figure S14 — Schematic diagram of repeat enrichment analyses. To identify neo-Y specific enrichment of repeat sequences, we counted the ratio of mate-pairs where one read spanned a neo-X/Y diagnostic SNP and the other read mapped to a repeat sequence in our consensus repeat library for D. miranda. (TIF) [file pbio.1001711.s015.tif]

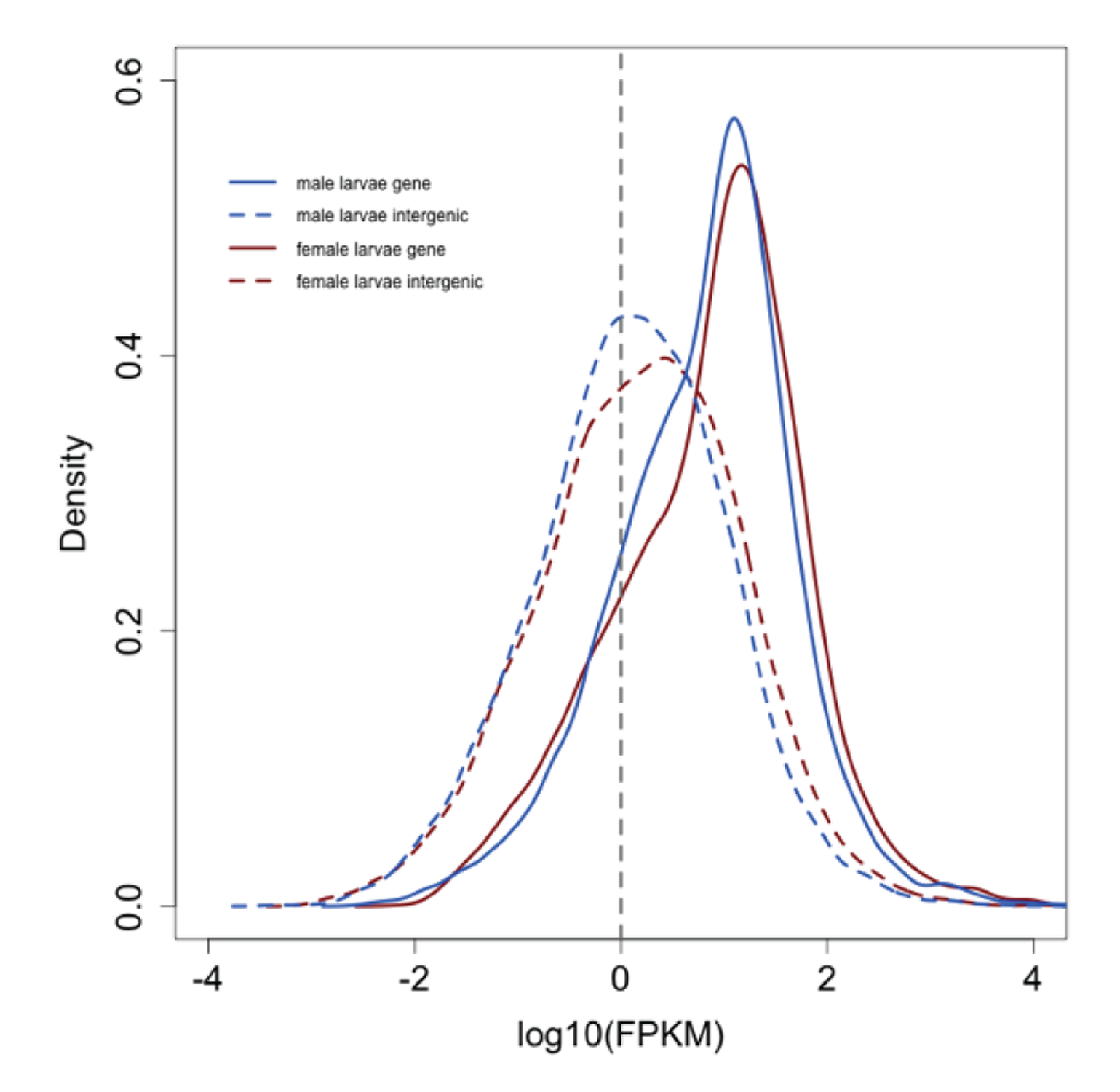

Supplement: Figure S15 — Identification of active and silent neo-Y genes. Shown is the histogram of FPKM values derived from genes (solid line) and intergenic regions (dotted line). The peak of the FPKM distribution at intergenic regions is chosen as a cut-off to determine whether a gene is active or silent on the neo-Y. (TIF) [file pbio.1001711.s016.tif]
