# Supplementary material for: The Epigenome of Evolving Drosophila Neo-Sex Chromosomes: Dosage Compensation and Heterochromatin Formation
Source: PLoS Biol. 2013 Nov 12;11(11):e1001711. doi: 10.1371/journal.pbio.1001711 (PMC3825665; doi:10.1371/journal.pbio.1001711)
Supplement: Table S1 — MSL-binding and H4K16ac enrichment for genes on different chromosomes. (DOCX) [file pbio.1001711.s017.docx]

**Supplementary Table 1.** MSL-binding and H4K16ac enrichment for genes on different chromosomes.

|  | MSL+/  H4K16ac- | MSL+/  H4K16+ | MSL-/  H4K16ac+ | dosage compensated | MSL-/H4K16ac-  (not compensated) | total |
| --- | --- | --- | --- | --- | --- | --- |
| chrXL | 28 | 766 | 389 | 1183 | 1090 | 2273 |
| chrXR | 34 | 1238 | 774 | 2046 | 1113 | 3159 |
| neo-X | 62 | 545 | 596 | 1203 | 1506 | 2709 |
| autosomes | 0 | 0 | 52 | 52 | 6627 | 6679 |
