# Supplementary material for: The Epigenome of Evolving Drosophila Neo-Sex Chromosomes: Dosage Compensation and Heterochromatin Formation
Source: PLoS Biol. 2013 Nov 12;11(11):e1001711. doi: 10.1371/journal.pbio.1001711 (PMC3825665; doi:10.1371/journal.pbio.1001711)
Supplement: Table S2 — GO terms significantly enriched in neo-X genes that are targeted/not targeted by the dosage compensation complex. (DOCX) [file pbio.1001711.s018.docx]

**Supplementary Table 2**. GO terms significantly enriched in neo-X genes

| GO term id | GO domain | GO term name |
| --- | --- | --- |
| GO terms significantly enriched in dosage compensated neo-X genes | | |
| GO:0032502 | biological_process | developmental_process |
| GO:0044707 | biological_process | single-multicellular_organism_process |
| GO:0051603 | biological_process | proteolysis_involved_in_cellular_protein_catabolic_process |
| GO:0019953 | biological_process | sexual_reproduction |
| GO:0006032 | biological_process | chitin_catabolic_process |
| GO:0007017 | biological_process | microtubule-based_process |
| GO:1901575 | biological_process | organic_substance_catabolic_process |
| GO:0030163 | biological_process | protein_catabolic_process |
| GO:0046716 | biological_process | muscle_cell_homeostasis |
| GO:0009057 | biological_process | macromolecule_catabolic_process |
| GO:0009056 | biological_process | catabolic_process |
| GO:0009987 | biological_process | cellular_process |
| GO:0044446 | cellular_component | intracellular_organelle_part |
| GO:0032991 | cellular_component | macromolecular_complex |
| GO:0005623 | cellular_component | cell |
| GO:0044422 | cellular_component | organelle_part |
| GO:0043226 | cellular_component | organelle |
| GO:0044464 | cellular_component | cell_part |
| GO:0001882 | molecular_function | nucleoside_binding |
| GO:0008135 | molecular_function | translation_factor_activity,_nucleic_acid_binding |
|  |  |  |
| GO terms significantly enriched in genes not dosage compensated on the neo-X | | |
| GO:0050794 | biological_process | regulation_of_cellular_process |
| GO:0060255 | biological_process | regulation_of_macromolecule_metabolic_process |
| GO:0044699 | biological_process | single-organism_process |
| GO:0050896 | biological_process | response_to_stimulus |
| GO:0019438 | biological_process | aromatic_compound_biosynthetic_process |
| GO:0009889 | biological_process | regulation_of_biosynthetic_process |
| GO:0051171 | biological_process | regulation_of_nitrogen_compound_metabolic_process |
| GO:0006508 | biological_process | proteolysis |
| GO:0010468 | biological_process | regulation_of_gene_expression |
| GO:0018130 | biological_process | heterocycle_biosynthetic_process |
| GO:0031326 | biological_process | regulation_of_cellular_biosynthetic_process |
| GO:0080090 | biological_process | regulation_of_primary_metabolic_process |
| GO:0015837 | biological_process | amine_transport |
| GO:0034654 | biological_process | nucleobase-containing_compound_biosynthetic_process |
| GO:0032774 | biological_process | RNA_biosynthetic_process |
| GO:0010556 | biological_process | regulation_of_macromolecule_biosynthetic_process |
| GO:0031323 | biological_process | regulation_of_cellular_metabolic_process |
| GO:0032501 | biological_process | multicellular_organismal_process |
| GO:0016070 | biological_process | RNA_metabolic_process |
| GO:0055085 | biological_process | transmembrane_transport |
| GO:0030182 | biological_process | neuron_differentiation |
| GO:0050789 | biological_process | regulation_of_biological_process |
| GO:0044271 | biological_process | cellular_nitrogen_compound_biosynthetic_process |
| GO:0006351 | biological_process | transcription,_DNA-dependent |
| GO:0030030 | biological_process | cell_projection_organization |
| GO:0019222 | biological_process | regulation_of_metabolic_process |
| GO:1901362 | biological_process | organic_cyclic_compound_biosynthetic_process |
| GO:2000112 | biological_process | regulation_of_cellular_macromolecule_biosynthetic_process |
| GO:0065007 | biological_process | biological_regulation |
| GO:0016020 | cellular_component | membrane |
| GO:0044425 | cellular_component | membrane_part |
| GO:0043234 | cellular_component | protein_complex |
| GO:0022892 | molecular_function | substrate-specific_transporter_activity |
| GO:0005549 | molecular_function | odorant_binding |
| GO:0005372 | molecular_function | water_transmembrane_transporter_activity |
