# Supplementary material for: The Epigenome of Evolving Drosophila Neo-Sex Chromosomes: Dosage Compensation and Heterochromatin Formation
Source: PLoS Biol. 2013 Nov 12;11(11):e1001711. doi: 10.1371/journal.pbio.1001711 (PMC3825665; doi:10.1371/journal.pbio.1001711)
Supplement: Table S3 — ChIP-seq reads mapped to neo-X and neo-Y specific variants, and undifferentiated neo-sex linked regions. (DOCX) [file pbio.1001711.s019.docx]

**Supplementary Table 3.** ChIP-seq reads mapped to neo-X and neo-Y specific variants, and undifferentiated neo-sex linked regions.

|  | H4K16ac- | H3K9me3 | Input |
| --- | --- | --- | --- |
| neo-X | 2616959 | 1898318 | 1623828 |
| neo-Y | 781891 | 1221193 | 762854 |
| common | 1327023 | 1782427 | 998135 |
| total mapped | 4725873 | 4901938 | 3384817 |
